# Associations between dietary habits, socio-demographics and gut microbial composition in adolescents

**DOI:** 10.1017/S0007114523002271

**Published:** 2024-03-14

**Authors:** Keri M. Kemp, Catheryn A. Orihuela, Casey D. Morrow, Suzanne E. Judd, Retta R. Evans, Sylvie Mrug

**Affiliations:** 1 Cardio-Renal Physiology and Medicine, Division of Nephrology, Department of Medicine, Heersink School of Medicine, University of Alabama at Birmingham, Birmingham, AL 35294, USA; 2 Department of Psychology, University of Alabama at Birmingham, Birmingham, AL, USA; 3 Department of Cell, Developmental and Integrative Biology, Heersink School of Medicine, University of Alabama at Birmingham, Birmingham, AL, USA; 4 Department of Biostatistics, School of Public Health, University of Alabama at Birmingham, Birmingham, AL, USA; 5 Department of Human Studies, School of Education, University of Alabama at Birmingham, Birmingham, AL, USA

**Keywords:** Eating patterns, Dietary pattern, Processed foods, Adolescent Diet Study, Rapid Eating Assessment for Participants Short Version

## Abstract

The relationship between dietary habits and microbiota composition during adolescence has not been well examined. This is a crucial knowledge gap to fill considering that diet–microbiota interactions influence neurodevelopment, immune system maturation and metabolic regulation. This study examined the associations between diet and the gut microbiota in a school-based sample of 136 adolescents (*M*_age_ = 12·1 years; age range 11–13 years; 48 % female; 47 % Black, 38 % non-Hispanic White, 15 % Hispanic or other minorities) from urban, suburban and rural areas in the Southeast USA. Adolescents completed the Rapid Eating Assessment for Participants and provided stool samples for 16S ribosomal RNA gene sequencing. Parents reported their child and family socio-demographic characteristics. The associations between diet and socio-demographics with gut microbiota diversity and abundance were analysed using multivariable regression models. Child race and ethnicity, sex, socio-economic status and geographic locale contributed to variation within microbiota composition (*β*-diversity). Greater consumption of processed meat was associated with a lower microbial *α*-diversity after adjusting for socio-demographic variables. Multi-adjusted models showed that frequent consumption of nutrient-poor, energy-dense foods (e.g. sugar-sweetened beverages, fried foods, sweets) was negatively associated with abundances of genera in the family *Lachnospiraceae* (*Anaerostipes*, *Fusicatenibacter* and *Roseburia*), which are thought to play a beneficial role in host health through their production of short-chain fatty acids (SCFAs). These results provide new insights into the complex relationships among socio-demographic factors, diet and gut microbiota during adolescence. Adolescence may represent a critical window of opportunity to promote healthy eating practices that shape a homoeostatic gut microbiota with life-long benefits.

The gut microbial community plays a crucial role in human physiology, and there is a growing appreciation for microbiome-based interventions in a wide spectrum of diseases^(1–3)^. To harness the gut microbiota for therapeutic applications, it is necessary to characterise the dramatic microbial community changes that occur across the life course from birth to old age^(4)^. It is well understood that the gut is colonised by microbiota during infancy, followed by a period of rapid microbial diversity expansion after the cessation of breast- or bottle-feeding and transition to solid foods^(5–7)^. A large body of research has shown how mode of delivery, gestational age, longevity of breast-feeding and antibiotics moderate this colonisation process^(5–8)^. Later in the lifespan, investigations have focused on how diet and lifestyle shape the gut microbiota in adulthood^(9,10)^. However, considerably less is known about the gut microbiome in the period between early childhood and adulthood. While it has been generally accepted that the infant microbiota reaches a stable adult-like state within the first 3 years of life^(6,7)^, new evidence indicates that the development of the gut microbiota continues through childhood^(11)^ and early adolescence^(12)^. However, few studies have examined how diet and other environmental factors shape the gut microbiota during adolescence^(13)^. This is a crucial knowledge gap to fill considering the important roles of diet–microbiota interactions in neurodevelopment, immune system maturation and metabolic regulation during adolescence^(14–16)^.

Adolescence marks a developmental period of increasing autonomy from caregivers in many cognitive, social and behavioural domains, including food acquisition, preparation and consumption^(17,18)^. Adolescent eating behaviours are shaped by a variety of factors, such as food availability, peer influences, socio-economic status (SES) and personal and cultural beliefs^(19)^. The overall quality of US adolescents’ diet is poor^(20)^, with few adolescents meeting the US Department of Agriculture recommended daily intake of fruits and vegetables^(21)^. These trends are not unique to the USA, with longitudinal studies from other Western countries also finding decreasing intake of fruits and vegetables and increasing consumption of sugary drinks during adolescence^(22)^. Diet quality is further negatively impacted by skipping breakfast^(23)^, which becomes more common in older youth^(24)^. Skipping breakfast is associated not only with a poorer diet quality but also with higher weight, other metabolic and cardiovascular risk factors and worse mental health among adolescents^(25)^. However, links between skipping breakfast and gut microbiota of adolescents have not been examined.

Dietary behaviours in adolescence are likely to impact health later in life^(26,27)^. Risk factors for diabetes and CVD in adolescence can predict adult health outcomes^(28,29)^. Although diet quality improves somewhat from adolescence to adulthood, the overall intake of the recommended macronutrients in young adulthood remains sub-optimal^(30)^. Thus, eating patterns developed during adolescence may carry long-lasting health consequences. However, little is known about how dietary intake during adolescence affects the still developing gut microbiota^(31,32)^.

## Study aims

To address these knowledge gaps, this study examines associations among nutritional intake, eating behaviours, urban–rural locale classifications and SES with the gut microbiome in a community sample of adolescents. The overall goal of this study is to provide insights into diet-related gut microbiota associations during adolescence, a crucial period of rapid physiological and neurological development that sets the stage for long-term health outcomes.

## Methods

### Sample

This study included early adolescents participating in Wave 1 of the Adolescent Diet Study, which examined the role of diet in adolescent health. Students in their first year of middle school attendance were recruited in 2019 from fifteen schools in urban, suburban and rural locations around Birmingham, Alabama, USA. The Generalizer program (www.thegeneralizer.org) was used to specify the target population, stratify the population and develop a sampling plan, including a list of schools for recruitment^(33,34)^. Stratification was based on variables at the school level (including proportion female, proportion White, Black, Asia, and Hispanic, proportion free and reduced lunch and urbanicity) and district level (including mean family income and education level). Thus, the fifteen schools in this study were selected to represent the socio-demographic characteristic of the state of Alabama. A total of 137 participants who were recruited in Wave 1 completed the dietary survey and provided a stool sample (online Supplementary Fig. 1). One participant was not included in the analyses due to insufficient sequencing depth of the faecal sample (< 5000 sequence reads), bringing the final number of participants included in this study to 136.

### Procedure

Trained project staff presented information about the study to the students in their classrooms and distributed packets containing information about the study and consent forms. Signed parent consent and student assent forms were collected at school approximately 1 week later (45 % participation rate). All data collection activities occurred at school during a regular school week. Trained research staff performed anthropometric measurements of students’ height and weight using a stadiometer and scale, respectively. Students completed a battery of self-report measures using electronic tablets during a non-academic class session, which included a self-report of dietary intake. One primary caregiver of each child was sent an online survey that included questions about child and family socio-demographic characteristics. Children and parents were compensated with gift cards for their time, with children receiving additional compensation for providing a stool sample. This study was conducted according to the guidelines laid down in the Declaration of Helsinki, and all procedures involving human subjects/patients were approved by the University of Alabama at Birmingham Institutional Review Board (IRB 300002344). Written informed consent/assent was obtained from all subjects.

#### Stool sample collection

Collection of stool samples in a non-clinical sample of adolescents is challenging, as many individuals find the process off-putting and embarrassing. This study utilised the wipe-based stool collection method, which streamlines the collection process and has been validated against other common collection procedures^(35)^. Participants collected a stool sample at home using a standardised wipe method previously described^(36)^. After a bowel movement, participants were instructed to wipe with a provided pre-moistened wipe, fold the wipe in half, seal it within a specimen bag and place the sample in a conventional freezer (–20°C), which slows microbial growth that could bias the microbiome results. Preservatives have been shown to introduce biases towards the detection of specific micro-organisms^(37)^; therefore, immediate freezing after stool collection was utilised in this study. After freezing the sample overnight, students could choose to either hand in their sample to the study staff at school or mail it using provided postage paid envelope. The collected samples were transported on ice and stored in a –80°C freezer at the University of Alabama at Birmingham prior to sequencing.

#### Microbial DNA extraction and sequencing

Stool sample DNA was isolated with the Zymo Research Quick-DNA Fecal/Soil Microbe Kit (catalogue # D6010). After DNA standardisation, the V4 region of the 16S rRNA gene was amplified with the New England Biolabs LongAmp Taq PCR kit (catalogue # E5200S) using primers and barcodes with slight modifications from the original Caporaso primers^(38)^ as previously described^(36)^. Amplicon PCR products were resolved on agarose gels, purified using the Qiagen QIAquick Gel Extraction Kit (catalogue # 28704) and standardised by fluorometry using the Quant-iT™ PicoGreen™ dsDNA Reagent from Thermo Fisher Scientific (catalogue # P11495). Standardised amplicon libraries were pooled and submitted for 2 × 250 bp paired-end sequencing on the Illumina MiSeq System at the University of Alabama at Birmingham Genomics Institutional Research Core. All samples were sequenced on the same Illumina run to reduce batch effects.

#### Sequence pre-processing and quality control

Quality filtering, error estimation, merging of reads, dereplication, removal of chimeras and selection of amplicon sequence variants (ASV) were performed in the QIIME 2 platform (v2020.6.0)^(39)^ with the DADA2 plugin and default options (v2020.6.0)^(40)^. To reduce artefacts due to amplification and sequencing error, ASV that had a count of less than three in each of five samples were filtered from the dataset. DADA2 was used to assign taxonomy to the ASV with the 138.1 SILVA small subunit ribosomal RNA database^(41,42)^ and the Ribosomal Database Project’s Training Set 18 and 11.5 database release^(43,44)^. ASV that were classified as either mitochondria or chloroplasts were removed from the dataset. The R package phangorn v2.9.0^(45)^ was used to build a reference-free maximum likelihood phylogenetic tree with the General Time Reversible+Γ + I model from a multiple alignment of the ASV made with DECIPHER v2.22.0^(46)^. The resulting ASV and taxonomy tables and phylogenetic tree were then imported into R 4.1.2^(32)^ using phyloseq v1.38.0 for visualisations and statistical analyses^(47,48)^.

### Measures

#### Dietary intake

Traditional dietary assessment methods are often too difficult and time-consuming to administer in a school setting. Therefore, this study utilised the Rapid Eating Assessment for Participants Short Version (REAP-S) that has been designed to assess diet related to the US Dietary Guidelines^(49)^. The REAP-S takes the participant about 10 min to complete, is written at a 5th-grade reading level^(50)^ and has been validated against 24-h dietary recalls and other measures of diet quality, including the Healthy Eating Index-2010^(49,51)^. The first thirteen items of the REAP-S (provided in online Supplementary Table 1) were utilised to evaluate how often adolescents consumed common foods (whole grains, fruits, vegetables, dairy, low-processed meat, processed meat, fried foods, sweets, fats/oils and sugar-sweetened beverages) or exhibited unhealthy eating behaviours (skipping breakfast, eating meals from a sit-down or take-away restaurant). All items were coded on a 3-point scale (1 = rarely/never, 2 = sometimes, 3 = usually/often), with higher scores indicating greater consumption of a food or a greater frequency of dietary behaviour. To examine diet associations with microbiota, the thirteen individual REAP-S items were utilised. In addition, overall diet quality scores ranging from 13 to 39 were derived according to Segal-Isaccson *et al*.^(49)^, with higher scores indicating a greater diet quality. Z-scores of diet quality were calculated and used in statistical modelling. Internal reliability was assessed using Cronbach’s *α*, which assesses how closely items correlate with each other^(52)^. Strong correlation (*α* > 0·70) between items in a dietary questionnaire may not be required if each item is designed to assess different aspects of the diet^(53)^.

#### Socio-demographic characteristics

Parents reported their child’s sex (0 = male; 1 = female) and race and ethnicity, which was categorised as non-Hispanic White (set as the reference), Black, Hispanic or other minority. Parents also reported annual household income (13-point scale from 1 = < $5000 per year to 13 > $90 000 per year) and their highest education level attained (7-point scale from 1 = no high school diploma to 7 = graduate or professional degree). A composite SES variable was created as the average of standardised household income and parental education (*r* = 0·51, *P* < 0·001). The geographic locale of the school site was obtained from the National Center for Education Statistics (NCES; http://nces.ed.gov/ccd/elsi/, accessed 23 June 2022) and categorised as rural (set as the reference), suburb or city. NCES locale classifications are based on population size or proximity to populated areas determined by the US Census Bureau and are assigned to schools based on their reported physical address location. School geographic locale correlates well with the environment where children reside and complete their daily routines. Missing data for demographic covariates were imputed using the expectation–maximisation algorithm in SAS 9.4.

#### BMI

Children were asked to remove shoes and bulky jackets or sweaters before anthropometric measurements. Two readings were taken for each weight and height to the nearest 0·01 kg or 0·10 cm. If the two readings differed by more than 0·20 kg or 0·50 cm, a third reading was taken. The two closest values were then averaged. BMI was calculated using WHO References 2007 SPSS macro package to calculate age and sex-corrected zBMI scores^(54)^. Sex-specific BMI-for-age percentiles were calculated based on the Centers for Disease Control and Prevention (CDC) Children’s BMI Tool for Schools. Then, BMI-for-age weight status categories were assigned according to the CDC definitions for underweight (less than the 5th percentile), healthy weight (5th percentile to less than the 85th percentile) and overweight or obese (equal to or greater than the 85th percentile).

#### Microbial *α*- and *β*-diversity

Diversity measurements are sensitive to differences in amplicon sequencing library sizes. Rarefying is a statistical tool that can successfully be implemented for diversity analyses when applied over multiple iterations^(55)^. The Multiple Iterations of Rarefying for Library Normalisation (mirlyn) R package v1.3.0 was used to rarefy libraries to the minimum sample size (24 341 sequences) over 1000 iterations. Then, *α*-diversity was estimated over the rarified matrix for Shannon Diversity – based on the number of taxa in a given community (richness) and their relative abundance, Simpson Evenness – a measure of how evenly individuals are distributed among taxa in a given community and Faith Phylogenetic Diversity – which represents the sum of the branch lengths of the phylogenetic tree linking all taxa in a given community. *β*-diversity, or the relative compositional similarity and differentiation among samples, was estimated with the vegan package v2.6.2 for rarified ASV tables after applying a Hellinger transformation^(56)^. *β*-diversity was calculated for Jaccard similarity, which is based only on the presence/absence of taxa, and Bray–Curtis similarity, which is weighted by taxon abundances.

### Data analyses

### Preliminary analyses

Preliminary analyses were conducted to examine the association of each socio-demographic variable (sex, race and ethnicity, geographic locale and SES) and zBMI with aspects of diet and microbial diversity. Bivariate associations of continuous demographic variables with diet variables and microbial *α-diversity* were tested using Pearson’s correlations using the cor.test function in the base R stats package. The associations with categorical variables were assessed with Wilcoxon rank sum tests or Kruskal–Wallis rank sum tests followed by post-hoc Wilcoxon rank sum tests with Benjamini–Hochberg false discovery rate corrections. Bivariate associations between *β*-diversity similarities and demographic variables were tested using permutational analysis of variance (PERMANOVA) and 999 permutations with the ‘adonis2’ function in vegan^(56)^. PERMANOVA measures the percentage of variation in microbial composition explained by the factors tested.

### Main analyses

#### Multivariable analyses of microbial diversity

The associations between dietary variables and microbial diversity were examined with multivariable general linearised models for *α*-diversity and multivariable PERMANOVA for *β*-diversity that simultaneously adjusted for zBMI, sex, race and ethnicity, geographic locale and SES. Eta-squared (*R* ^2^) values indicated the relative explanatory value that each dietary or socio-demographic variable had on the variance in *β*-diversity. The associations of socio-demographic variables with *β*-diversity were visualised with unconstrained principal coordinate analysis of Bray–Curtis distances utilising the R packages ggfortify v.4.14 and ggplot2 v3.3.6. The ‘envfit’ function from the vegan package was used to fit the covariate vectors onto a 2D ordination of the first two principal coordinate components with 999 permutations. *P*-values calculated from ‘envfit’ were adjusted by the false discovery rate method for multiple comparison.

#### Microbial taxon differential abundance

Differential abundance testing was conducted on ASV count data with the R package Microbiome Multivariable Associations with Linear Models (MaAsLin2 v.1.8.0)^(57)^. ASV were first summarised at the genus, family and phylum taxonomic levels using the tax_glom function in phyloseq. Then, data were scaled by the trimmed mean of M-values method^(58)^, which controls well for false discovery rate with datasets that exhibit a high degree of asymmetry and sparsity, like microbiome data in which a few taxa are abundant while most are rare. The trimmed mean of M-values scaling factor is calculated as the weighted mean of log-ratios between each pair of samples, after excluding the highest count ASV and ASV with the largest log-fold change. The normalisation factors for each sample are the product of the trimmed mean of M-values scaling factor and the original library size. MaAsLin2 was implemented using negative binomial models, which adjusted for sex, zBMI, race and ethnicity, locale and SES with a Benjamini–Hochberg false discovery rate correction (*q* = 0·15). Only taxa with non-zero values in at least 75 % of the study samples (min_prevalence = 0·75) were tested in differential abundance models. Microbial differential abundance was visualised with ggplot2 using the geom_tile() function.

## Results

### Study sample

Characteristics of the 136 adolescents included in this study are provided in Table 1. The median participant age at the time of stool sample collection was 12 years (range, 11–13 years). The study sample was 48 % female and had a racial and ethnic breakdown of 47 % Black, 38 % non-Hispanic White, 9 % Hispanic and 6 % other racial and ethnic minorities. Most participants (53 %) were overweight or obese, while 45 % had normal BMI and 2 % were underweight. The sample was socio-economically heterogeneous, with a median family income of $40 000–50 000 and median parent education being ‘some college’, and closely mirrored the demographic composition of the sampled area.

**Table 1.** Characteristics of the Adolescent Diet Study cohort (Numbers and percentages; mean values and standard deviations)

	*n* 136	
Characteristics	*n*	%
Age, years		
Mean	12·1	
Range	11–13	
zBMI		
Mean	1·2	
sd	1·5	
Underweight (<5th percentile)	3	2
Normal BMI (5th–85th percentile)	61	45
Overweight or obese (≥85th percentile)	72	53
Sex, female	65	48
Race and ethnicity		
Non-Hispanic White	52	38
Black	64	47
Hispanic	12	9
Other racial and ethnic minorities	8	6
School locale		
Rural	18	13
Suburban	86	63
City	32	24
Parent education		
<12th grade, no diploma	14	12
High school graduate/GED	20	18
Some college no degree	27	24
Technical school/Associate degree	19	17
Bachelor’s degree	20	18
Graduate or professional degree	4	4
Missing	8	7
Household income per year, Median	$40 000–50 000	

zBMI, standardised BMI; GED, General Education Diploma.

### Diet and demographics

Ninety-three percentage (*n* 126) of the study sample completed all thirteen questions on the dietary survey. The associations between diet and socio-demographic variables are presented in Table 2. The estimate of the internal consistency of the overall REAP-S scale as measured by Cronbach’s *α* was 0·60, similar to the reported values of other studies utilising the REAP-S^(59,60)^ and similar brief dietary questionnaires^(53)^. Black adolescents had reduced overall diet quality measured by the REAP-S survey compared with non-Hispanic White and Hispanic adolescents (*P* < 0·05). Black adolescents were also more likely to skip breakfast compared with White adolescents (*P* < 0·05). Higher SES was linked to greater consumption of meat and fats (*r* = 0·20, *P* = 0·02 and *r* = 0·17, *P* = 0·048, respectively), lower consumption of sugary drinks (*r* = 0·17, *P* = 0·048) and lower frequency of skipping breakfast (*r* = –0·22, *P* = 0·01). Children in suburban locales consumed more meat compared with those in city and rural locales (*P* < 0·05).

**Table 2.** Dietary associations with demographics (Numbers; mean values and standard deviations)

		Sex					Race and ethnicity									Locale							SES		zBMI	
Diet variable*	*n*	Male‡		Female‡		*P* §	Non-Hispanic White‡		Black‡		Hispanic‡		Other minority‡		*P* ||	Rural‡		Suburb‡		City‡						
		Mean	sd	Mean	sd		Mean	sd	Mean	sd	Mean	sd	Mean	sd		Mean	sd	Mean	sd	Mean	sd	*P* ||	*r*	*P* ¶	*r*	*P* ¶
Diet quality Z-score	134	1·8	0·3	1·8	0·3	0·68	1·8	0·3^A^	1·7	0·3^B^	2·0	0·3^A^	1·8	0·4^AB^	0·006	1·7	0·3	1·8	0·3	1·7	0·2	0·60	–0·04	0·61	0·05	0·54
Skipping breakfast	134	1·7	0·7	1·9	0·8	0·051	1·5	0·7^A^	2·0	0·7^B^	1·8	0·8^AB^	2·0	0·8^AB^	<0·001	1·7	0·9	1·8	0·7	1·9	0·7	0·59	–0·20	0·02	0·07	0·40
Eating out	133	1·8	0·6	1·7	0·7	0·44	1·7	0·5	1·9	0·8	1·5	0·7	1·4	0·5	0·08	1·7	0·5	1·7	0·7	2·0	0·7	0·15	–0·03	0·71	–0·20	0·02
Whole grains	134	1·8	0·7	2·0	0·6	0·03	1·9	0·7	1·9	0·6	2·0	0·6	1·9	0·8	0·96	2·0	0·5	2·0	0·7	1·8	0·7	0·47	0·15	0·09	0·00	0·98
Fruits	134	1·9	0·8	2·0	0·7	0·49	1·8	0·7	2·0	0·7	1·8	0·8	1·9	0·6	0·32	2·2	0·9	1·8	0·7	2·0	0·6	0·10	–0·14	0·11	0·09	0·28
Vegetables	135	2·4	0·7	2·5	0·6	0·24	2·4	0·8	2·4	0·6	2·6	0·7	2·6	0·5	0·72	2·4	0·7	2·4	0·7	2·5	0·6	1·0	–0·06	0·46	0·06	0·46
Dairy/cheese	132	1·9	0·8	1·9	0·7	0·61	1·9^A^	0·8	2·0^A^	0·7	1·8^A^	0·7	1·3^B^	0·5	0·04	2·1	0·8	1·8	0·7	2·0	0·8	0·34	0·09	0·28	–0·03	0·73
Meat	134	2·1	0·6	2·1	0·7	0·73	2·2	0·6	2·1	0·7	1·8	0·6	2·0	0·8	0·42	1·8^A^	0·5	2·2^B^	0·6	1·9^A^	0·7	0·002	0·20	0·02	0·01	0·91
Processed meats	133	2·1	0·6	2·0	0·7	0·37	2·0	0·6	2·1	0·7	1·6	0·5	2·2	0·7	0·07	2·1	0·7	2·0	0·6	2·1	0·7	0·40	0·07	0·40	–0·05	0·55
Fried foods	134	2·1	0·7	2·0	0·8	0·66	2·0	0·7	2·1	0·8	1·6	0·5	1·9	0·8	0·10	2·1	0·8	2·0	0·7	2·1	0·7	0·51	<0·01	0·96	–0·08	0·35
Fatty snacks (chips)	133	2·3	0·6	2·1	0·7	0·30	2·3	0·6	2·2	0·7	1·9	0·5	1·9	0·8	0·19	2·3	0·6	2·2	0·7	2·1	0·6	0·34	0·11	0·21	–0·04	0·64
Fats (oil, butter)	132	2·0	0·8	2·0	0·8	0·81	1·9	0·8	2·1	0·8	1·7	0·9	2·4	0·5	0·12	2·1	0·9	2·0	0·8	2·2	0·7	0·52	0·17	0·049	–0·02	0·79
Fatty sweet products	133	2·0	0·7	1·9	0·7	0·50	1·9	0·7	2·1	0·7	1·7	0·7	1·8	0·7	0·23	1·9	0·5	2·0	0·7	2·0	0·8	0·90	0·04	0·67	–0·13	0·13
Sodas/sugary drinks	132	2·0	0·7	2·0	0·8	0·96	1·8	0·7	2·1	0·7	1·8	0·8	2·4	1·0	0·03†	1·9	0·7	1·9	0·8	2·1	0·7	0·35	–0·22	0·01	0·01	0·88

SES, socio-economic status; zBMI, standardised BMI.

* Diet variables were scored on a 3-point scale as 1 = rarely/never, 2 = sometimes and 3 = usually/often consume a food type or exhibit an eating behaviour. Supercript letters indicate statistical groupings based on Wilcoxon rank sum and Kruskal–Wallis rank sum tests at *α* = 0.05.

† No significant pair-wise tests.

‡ Mean (sd).

§ Wilcoxon rank sum tests.

|| Kruskal–Wallis rank sum tests.

¶ Pearson correlations.

### Microbiome sequencing

Regarding microbiota, 96·7 % (∼6·57 million) of sequences reads were retained in the dataset after filtering rare taxa and those classified as mitochondria or chloroplasts. Rarefying to the minimum sample size of 24, 341 reads sufficiently controlled for variation in sequence depth, which was not associated with *α*- or *β*-diversity (*P* > 0·05 in all cases; online Supplementary Tables 2 and 5). The five most abundant phyla in decreasing order were Bacillota (formally Firmicutes; mean relative abundance: 0·57 (sd 0·15)), Bacteroidota (0·32 (sd 0·16)), Actinomycetota (formally Actinobacteriota; 0·05 (sd 0·07)), Pseudomonadota (formally Proteobacteria; 0·03 (sd 0·09)) and Verrucomicrobiota (0·02 (sd 0·04)) (online Supplementary Fig. 2).

### Microbial *β*-diversity

**Fig. 1.**
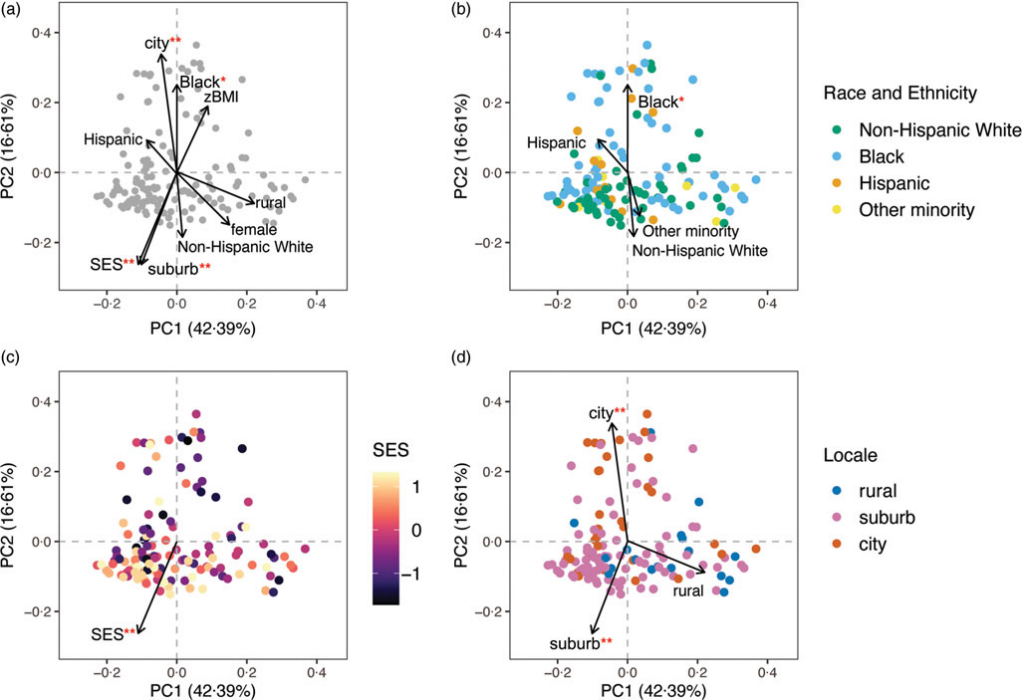
*β*-diversity or the inter-individual variation in gut microbiota composition represented by unconstrained principal coordinate analysis (PCoA) of the Bray–Curtis distance. Global structure (a) of gut microbiota composition and grouping patterns based on (b) race and ethnicity with ‘non-Hispanic White’ as the reference, (c) socio-economic status (SES) and (d) geographic locale with ‘rural’ as the reference are shown. Each point represents an individual from the study sample (*n* 136 for all panels) and individuals whose points are closer together have more similar gut microbiota composition. Vector arrows indicate the direction of gradient for covariates and were obtained via the vegan R package envfit function and are scaled by the squared correlation, *R* ^*2*^, from 999 permutations fitting each value of the covariate to the 2D ordination space. Percentages on the axes represent the proportion of variation explained by the two first principal coordinates (PC) of the PCoA. Significance of permutation tests after applying a false discovery rate correction is denoted as ‘**’ for q < 0·01 and ‘*’ for q < 0·05.

Similarity in gut microbial composition, or *β*-diversity, was evaluated using Jaccard distances, based on taxon presence/absence, and Bray–Curtis distances, based on weighting taxon abundance. The percentage of variation in *β*-diversity explained by each demographic variable was examined with PERMANOVA (online Supplementary Table 2). Multivariable PERMANOVA revealed that adolescent race and ethnicity, SES and geographic locale were associated with both Bray–Curtis and Jaccard *β*-diversity and together explained around 7 % of the total variation in microbial composition. Sex explained 1 % of the variation in Jaccard similarity, while zBMI was not statistically associated with either *β*-diversity metric. Figure 1 presents the unconstrained principal coordinate analysis of Bray–Curtis similarity fitted with demographic variable vectors. None of the thirteen dietary variables was associated with *β*-diversity in multivariable PERMANOVA after adjusting for SES, race and ethnicity, geographic locale, sex and zBMI (online Supplementary Table 3).

### Microbial *α*-diversity

**Fig. 2.**
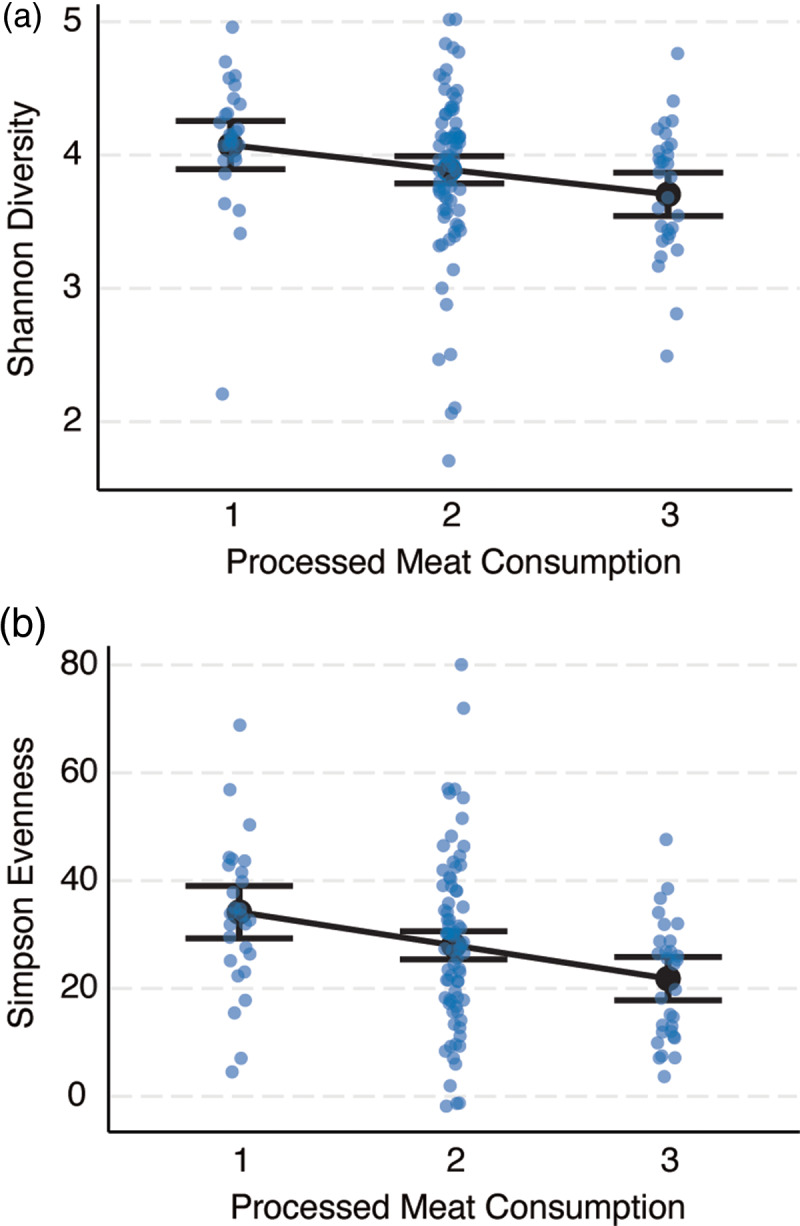
Processed meat consumption is negatively correlated with two metrics of microbial *α*-diversity, (a) Shannon Diversity (*n* 133, *β* = –0·19, se = 0·08, *P* = 0·03) and (b) Inverse Simpson Evenness (*n* 133, *β* = –0·23, se = 2·05, *P* = 0·01). The REAP-S asked how often processed meat (e.g. bologna, salami, hotdogs, sausage) was consumed instead of low-processed meats (e.g. fish, poultry, red meat) in an average week and was scored on a 3-point scale (1 = rarely/never, 2 = sometimes, 3 = usually/often). Partial residual plots are shown for general linearised models adjusting for the effects of *sex* (female/male), *race and ethnicity* (non-Hispanic White, Black, Hispanic, other minority), standardised BMI (zBMI), socio-economic status (SES) and geographic locale (rural, suburb, city). Error bars depict the 95 % CI of the predicted estimates.

Bivariate associations between demographic variables and *α*-diversity indices are displayed in online Supplementary Table 4. In summary, zBMI was negatively correlated with Simpson Evenness (*r* = –0·20, *P* = 0·02) and SES was negatively correlated with Faith Phylogenetic Diversity (*r* = –0·19, *P* = 0·03). The associations between diet variables and *α-Diversity* are shown in online Supplementary Table 5. In summary, general linearised models adjusting for SES, race and ethnicity, locale, sex and zBMI showed that greater consumption of processed meat was associated with lower Shannon Diversity (*β* = –0·19, se = 0·08, *P* = 0·03) and Inverse Simpson Evenness (*β* = –0·23, se = 2·05, *P* = 0·01; Fig. 2). Greater fruit consumption was associated with greater Faith Phylogenetic Diversity (*β* = 0·17, se = 1·03, *P* = 0·045).

### Microbial taxon differential abundance

**Fig. 3.**
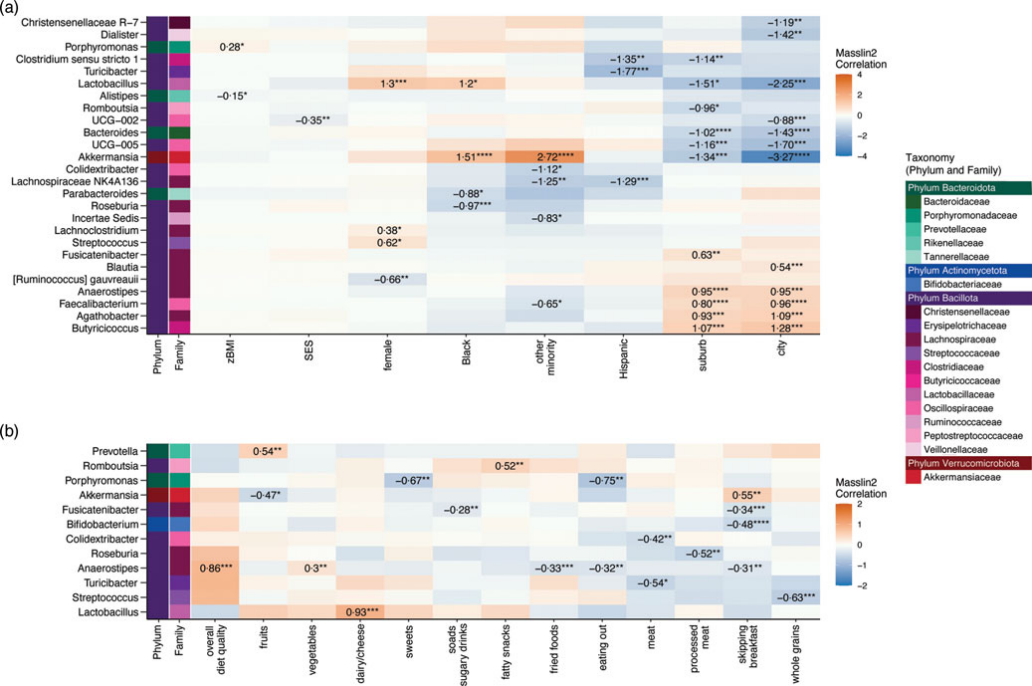
Associations between socio-demographic variables (*n* 136 participants) and (b) dietary variables (*n* 132–135 participants) and the abundance of bacterial taxa at the genus level using Microbiome Multivariable Associations with Linear Models (MaAsLin2, package on R). MaAsLin2 multi-adjusted for sex (male = reference), race and ethnicity (non-Hispanic White = reference group), standardised BMI (zBMI), socio-economic status (SES) and geographic locale (rural = reference group). Genera are displayed on the left y-axis and are colour-coded by the taxonomic family and phylum they belong to. The MaAsLin2 coefficient (effect size) is shown only for significant statistical associations after Benjamini–Hochberg false discovery rate correction (q = 0·15). The corrected significance is denoted as ‘****’ for q < 0·01, ‘***’ for q < 0·05, ‘**’ for q < 0·10 and ‘*’ for q < 0·15.

The statistically significant results from Microbiome Multivariate Association with Linear Models (MaAsLin2) examining the associations between dietary and socio-demographic variables and taxon abundance are shown at the genus level in Fig. 3 and at the family and phylum levels in online Supplementary Fig. 3. Greater consumption of processed meat was associated with lower abundance of the genus *Roseburia,* while more consumption of fried foods was associated with lower *Anaerostipes,* and greater intake of sodas/sugary drinks was associated with lower *Fusicatenibacter.* Frequently skipping breakfast was associated with greater abundance of the genus *Akkermansia* (as well as its family, *Akkermansiaceae*, and phylum, Verrucomicrobiota) and reduced abundance of the genera *Anaerostipes*, *Fusicatenibacter* and *Bifidobacterium*. Greater zBMI was statistically significantly associated with two genera within the phylum Bacteroidota, albeit in opposite directions, positive for *Porphyromonas* and negative for *Alistipes*. Full results for all MaAsLin2 results are presented in online Supplementary Table 6.

## Discussion

Despite the crucial importance of the gut microbiota for the development and regulation of the nervous, immune and metabolic systems, surprisingly little is known about how dietary intake and other environmental factors are associated with the gut microbiota composition during adolescence. This study addressed these gaps by examining relationships between diet and intake of major food groups with the gut microbiota of 136 adolescents (11–13 years of age). The main findings showed that socio-demographic characteristics (race and ethnicity, SES and geographic locale) explained a relatively large degree of microbial variation. Additionally, even after adjusting for BMI and socio-demographics, greater consumption of processed meat was related to lower microbial *α*-diversity, a hallmark of microbial dysbiosis, and greater intake of processed foods (sugary drinks and sodas, fried and fatty snacks, processed meats) and skipping breakfast were associated with reduced abundance of potentially beneficial taxa known to produce SCFA.

### Processed foods and SCFA-producing taxa

Greater consumption of processed meats was significantly associated with lower abundances of the genus *Roseburia*, which play an important role in the human gut by producing SCFA, such as acetate, propionate and butyrate^(61)^. Likewise, greater intake of fried foods and sodas/sugary drinks was associated with a reduction of the SCFA-producing bacteria *Anaerostipes* and *Fusicatenibacter*, respectively. Results indicate that greater consumption of processed foods may be associated with a reduced abundance of potentially beneficial taxa involved SCFA production.

The finding that intake of processed meat, fried foods and sodas/sugary drinks reduced the abundance of *Roseburia, Fusicatenibacter* and *Anaerostipes*, and other SCFA-producing taxa is particularly noteworthy given that *these* taxa have been identified as key players in gut homoeostasis through the regulation of immune cells, cytokine release and maintenance of the gut barrier function^(61,62)^. It is currently understood that childhood and adolescence represent a critical time when *Roseburia* and other SCFA-producing expand within the gut microbial community^(4)^. As evidence, a longitudinal study tracking gut microbial colonisation across the first years of life found that *Roseburia*, *Faecalibacterium* and other SCFA-producing taxa within the Family *Lachnospiraceae* were prevalent among children but had not yet reached adult levels of abundance by 5 years of age^(11)^. A greater intake of processed foods during adolescence may limit the degree or slow the timeline of expansion for key SCFA-producing taxa and warrants further investigation in longitudinal studies. Numerous studies have shown that food additives, including emulsifiers, non-energetic artificial sweeteners and preservatives, impact gut microbial diversity and composition^(63)^. Moreover, dietary emulsifiers can directly alter the expression of bacterial virulence genes and increase pathogenic interactions between the gut microbiota and the host^(64)^. Thus, a diet rich in processed foods may alter the landscape of microbial competition or the gut environment to either promote or inhibit the growth of certain bacterial types.

### Processed meat consumption

Greater processed meat consumption (e.g. hotdogs, bologna, salami, bacon) was also associated with an increased abundance of Pseudomonadota (formally Proteobacteria) independent of other study variables. Elevated Pseudomonadota abundance is considered a potential diagnostic signature of microbial dysbiosis and risk of non-communicable diseases^(65,66)^. Among the five major bacterial phyla in the gut, Pseudomonadota is the most unstable over time^(67)^ and has been considered a ‘first-responder’ to dietary and environmental changes^(65)^. A greater abundance of Pseudomonadota has been linked to metabolic disorder, inflammation and increased levels of the proinflammatory IL-17^(65,66)^. Multivariable models also revealed reduced diversity with increased processed meat intake. Interestingly, *α*-diversity was not related to consumption of low-processed red meat and poultry. These results are consistent with those from older adults, where consumption of high-processed meat was negatively associated with *α*-diversity while moderate consumption of low-processed red meat was positively associated with *α*-diversity^(68)^. Together, these findings suggest that the processing and preparation of animal protein, rather than its intake alone, may be an important mediator of gut microbial composition and diversity. Notably, higher intake of processed meat, but not total animal protein, has been associated with greater risk factors for cardiometabolic disease^(69)^. These results provide rationale for future study of the links between processed meat consumption, gut microbiota and markers of health during adolescence.

### Skipping breakfast

In this study, the frequency of breakfast consumption was not linked with microbial diversity in adolescents, contrasting with prior findings that more frequent breakfast consumption was associated with greater gut microbial diversity in adults^(70)^. However, this study found that adolescents who frequently skipped breakfast had lower abundances of the aforementioned SCFA-producers *Anaerostipes* and *Fusicatenibacter* as well as *Bifidobacterium.* Members of the genus *Bifidobacterium* are among the first microbes to colonise the gastrointestinal tract and are believed to exert positive health benefits^(71)^. Bifidobacteria produce lactate and acetate which other microbial taxa convert to butyrate and propionate. These microbial derived SCFA are believed to impact neural networks in the developing adolescent brain that are critical for normal cognitive, emotional and social functioning and development^(72)^. Interestingly, skipping breakfast was also associated with an increased abundance of the genus *Akkermansia*. *Akkermansia* has been reported as enriched in healthy individuals and is inversely associated with multiple diseases states including obesity, the metabolic syndrome and inflammatory bowel disease^(73)^. Further research is needed to understand how skipping breakfast may positively or negatively affect the gut microbiota.

### Socio-demographics and microbial composition (*β*-diversity)

An important strength of this study was the inclusion of participants of different races and ethnicities, SES and geographic locales. Previous investigations of diet associations with the gut microbiota during childhood have been conducted in samples with a relatively low degree of ethnic and socio-economic heterogeneity^(74–76)^. In this study, race and ethnicity, SES and geographic locale were each independently associated with adolescent gut microbial composition or *β-diversity*, while BMI was not. None of the diet variables examined in this study was independently associated with *β-diversity*. These findings are similar to a study, based on the American Gut Project, which found that posteriori eating patterns were more strongly associated with *β-diversity* than the intake of individual diet components^(77)^.

Long–term eating patterns may arise from interactions among SES, cultural practices and the local food environment within which adolescents reside and attend school^(78)^. Food environment dimensions include *availability*, or the adequacy of the supply of healthy food, *accessibility*, or the location of the food supply and ease of getting to that location, and *accommodation*, how well local food sources accept and adapt to local residents’ needs^(79)^. Both city and rural food environments can present barriers to access large supermarkets that typically have greater diversity of fresh foods^(80)^. Barriers to healthy eating in these areas include travel distance and transportation logistics, as well as the cost of healthy food items^(81,82)^. In this cohort, adolescents from less affluent families consumed more processed meat and more sodas/sugary drinks. In terms of geography, adolescents in rural and city locales consumed more processed meat compared with adolescents in the suburbs. Further research is needed to understand how the upstream effects of SES, culture and the local food environment influence dietary intakes of adolescents with downstream effects on the gut microbiota composition.

### Study limitations

This study focused on dietary habits during adolescence and did not capture information about early life events, such as duration of breast-feeding and gestational age, which are known to have lasting effects on the developing gut microbiota through childhood and early adolescence^(11,75,76)^. Though the age range of adolescents included in this study was narrow, pubertal status may have contributed to the inter-individual variability in microbial composition and future studies are needed to examine this in greater detail. A large number of study participants were categorised as overweight or obese, and although the diet–microbiota associations reported here may be reflective of patterns in the Deep South region of the USA, they may not be reflective of adolescent cohorts with lower proportions of obesity.

This study emphasised biological replicates and could not assess the degree of technical variation in the microbiome data through the use of technical replicates^(37)^. It is also possible that differences in sample transportation time to the University of Alabama at Birmingham campus are responsible for some variation in sequencing data. The use of 16s rRNA gene sequencing in this study provided a limited window of information and microbial function was inferred from taxonomy. Future studies would benefit from metagenomic sequencing to directly assess microbial function.

Additionally, there were benefits and limitations associated with using the REAP-S self-administered dietary questionnaire in this study^(49)^. The REAP-S provided sufficient information on multiple important food groups and could be easily completed within the school setting, thus supporting feasibility. However, it did not capture the intake of all nutrients and dietary behaviours that may be related to gut microbiota composition. Future studies should utilise 24-h diet recalls to derive detailed information on macro and micro-nutrients, as well as timing of food intake. Longitudinal studies, utilising multiple diet recalls and gut microbiota assessments over time, will provide additional insights into how dietary intakes affect the developing adolescent gut microbiome.

### Conclusion

Adolescence is a period when individuals gain more autonomy over their dietary intake and food choices, setting the stage for future health^(17)^. A main finding from this study is that greater intake of processed foods was associated with a decreased abundance of key SCFA-producing microbial taxa, and the consumption of processed meats, in particular, was associated with a significantly lower microbial diversity. These results provide new insight into diet–microbiota associations during adolescence, a time of transformative growth when dietary intake affects the maturation of multiple physiological systems.

## Supporting information

Kemp et al. supplementary material Kemp et al. supplementary material

## Supplementary material

For supplementary material referred to in this article, please visit https://doi.org/10.1017/S0007114523002271
