# Supplementary material for: Associations between dietary habits, socio-demographics and gut microbial composition in adolescents
Source: Br J Nutr. 2023 Oct 18;131(5):809–20. doi: 10.1017/S0007114523002271 (PMC10864997; doi:10.1017/S0007114523002271)
Supplement: Kemp et al. supplementary material [file S0007114523002271sup001.docx]

**Supplemental to:**

Kemp et al. *Associations between dietary habits, sociodemographics, and gut microbial composition in adolescents*

**Supplemental Table 1.** Twelve self-administered dietary questions derived from the Rapid Eating and Activity Assessment for Participants Short Version (Segal-Isaacson et al., 2004).

| **Category** | **In an average week…** |
| --- | --- |
| 1. Skipping breakfast | …how often do you skip breakfast? |
| 2. Eating out | …how often do eat 4 or more meals from a sit-down or take out restaurant? |
| 3. Whole grains | …how often do you eat less than 2 servings of whole grain products or high fiber starches a day?  These are some examples of what a serving means: 1 slice of 100% whole grain bread; 1 cup whole grain cereal like Shredded Wheat, Wheaties, Grape Nuts, high fiber cereals; oatmeal; 3–4 whole grain crackers; 1/2 cup of brown rice or whole wheat pasta; boiled or baked potatoes, yucca, yams, or plantain. |
| 4. Fruits | …how often do you eat less than 2 servings of fruit a day? These are some examples of what a serving means: 1/3 cup of fruit; 1 medium-sized fruit or 3/4 cup of 100% fruit juice. |
| 5. Vegetables | …how often do you eat less than 3-4 servings of vegetables/potatoes a day?  Serving = ½ cup vegetables/potatoes, or 1 cup leafy raw  vegetables |
| 6. Dairy/cheese | …how often do you eat less than 2 servings of milk, yogurt, or cheese a day?  These are some examples of what a serving means: 1 cup milk or yogurt; 1–2 ounces of cheese |
| 7. Meat | …how often do you eat more than 8 ounces (see sizes below) of meat, chicken, turkey, or fish per day?  These are some examples of what a serving means: 3 ounces of meat or chicken is the size of a deck of cards or ONE of the following: 1 regular hamburger, 1 chicken breast or leg (thigh or drumstick), or pork chop. |
| 8. Processed meats | …how often do you use regular processed meats (like bologna, salami, corned beef, hotdogs, sausage, or bacon) instead of low-fat processed meats (like roast beef, turkey, lean ham, low-fat cold cuts/hotdogs)? |
| 9. Fried foods | …how often do you eat fried foods such as fried chicken, fried fish, French fries, fried plantains, tostones or fried yucca? |
| 10. Fatty snacks | …how often do you eat regular potato chips, nacho chips, corn chips, crackers, regular popcorn, nuts, instead of pretzels, low-fat chips, or low-fat crackers, air popped popcorn? |
| 11. Fats/oils | …how often do you add butter, margarine, or oil to bread, potatoes, rice, or vegetables at the table? |
| 12. Fatty sweet products | …how often do you eat sweets like cake, cookies, pastries, donuts, muffins, chocolate, and candies, more than 2 times per day? |
| 13. Sugary drinks/sodas | …how often do you drink 16 ounces or more of non-diet soda, fruit drink/punch or Kool-Aid a day?  Note: 1 can of soda = 12 ounces |

**Supplemental Table 2.** Bray-Curtis and Jaccard β-diversity associations with demographic variables using univariable and multivariable permutational analysis of variance (PERMANOVA; 999 permutations; n=136).

|  | **Bray-Curtis** | | | | |  | **Jaccard** | | | | |
| --- | --- | --- | --- | --- | --- | --- | --- | --- | --- | --- | --- |
|  | **Univariable Models** | |  | **Multivariable Model** | |  | **Univariable Models** | |  | **Multivariable Model** | |
| **Variable** | ***R*²** | ***p*** |  | ***R*²** | ***p*** |  | ***R*²** | ***p*** |  | ***R*²** | ***p*** |
| Sequencing Depth | .008 | .34 |  | — | — |  | .008 | .38 |  | — | — |
| zBMI | .011 | .06 |  | .009 | .09 |  | .010 | **.049** |  | .009 | 0.11 |
| SES | .016 | .003 |  | .016 | **.004** |  | .013 | **.004** |  | .013 | **.005** |
| Sex, *Male** |  |  |  |  |  |  |  |  |  |  |  |
| *Female* | .011 | .048 |  | .011 | .05 |  | .010 | .06 |  | .48 | **.048** |
| Race/Ethnicity, *White** |  |  |  |  |  |  |  |  |  |  |  |
| *Black* | .018 | .003 |  | .014 | **.004** |  | .014 | **.001** |  | .012 | **.003** |
| *Hispanic* | .008 | .29 |  | .010 | .12 |  | .008 | .36 |  | .009 | 0.11 |
| *Other Minority* | .006 | .76 |  | .006 | .72 |  | .007 | .75 |  | .007 | 0.65 |
| Locale, *Rural** |  |  |  |  |  |  |  |  |  |  |  |
| *City* | .019 | .002 |  | .016 | **.004** |  | .015 | **.001** |  | .013 | **.003** |
| *Suburb* | .014 | .003 |  | .015 | **.003** |  | .012 | **.006** |  | .012 | **.006** |

Bolded values denote statistical significance at *p* < .05. Asterisks denote reference levels. Abbreviations: SES, socioeconomic status; zBMI, standardized body mass index

**Supplemental Table 3.** Associations between β-diversity similarities and dietary variables using PERMANOVAs adjusted for zBMI, sex, race/ethnicity, geographic locale, and SES (999 permutations; n=136).

|  | **Bray-Curtis** | | |  | **Jaccard** | | |
| --- | --- | --- | --- | --- | --- | --- | --- |
| **Variable (*n* missing)** | ***R*²** |  | ***p*** |  | ***R*²** |  | ***p*** |
| Skipping breakfast (2) | .008 |  | .35 |  | .007 |  | 0.44 |
| Eating out (3) | .008 |  | .24 |  | .008 |  | .24 |
| Whole grains (2) | .006 |  | .74 |  | .007 |  | .70 |
| Fruits (2) | .009 |  | .18 |  | .008 |  | .24 |
| Vegetables (1) | .006 |  | .64 |  | .007 |  | .65 |
| Dairy/cheese (4) | .008 |  | .25 |  | .008 |  | .30 |
| Meat (2) | .009 |  | .16 |  | .009 |  | .08 |
| Processed meats (3) | .009 |  | .16 |  | .009 |  | .14 |
| Fried foods (2) | .010 |  | .07 |  | .009 |  | .09 |
| Fatty snacks (3) | .007 |  | .47 |  | .007 |  | .45 |
| Fats/oils (4) | .009 |  | .22 |  | .008 |  | .29 |
| Fatty sweet products (3) | .009 |  | .20 |  | .008 |  | .22 |
| Sodas/sugary drinks (4) | .008 |  | .36 |  | .008 |  | .35 |

**Supplemental Table 4.** Bivariate associations between α-diversity indices and demographic variables

| **Variable** | **Shannon Diversity** | |  | **Simpson Index** | |  | **Faith’s PD** | |
| --- | --- | --- | --- | --- | --- | --- | --- | --- |
|  | ***r*** | ***p*-value** |  | ***r*** | ***p*-value** |  | ***r*** | ***p*-value** |
| Sequencing Depth | .05 | .53^1^ |  | .04 | .66^1^ |  | .10 | .25^1^ |
| zBMI | .16 | .07^1^ |  | **-.20** | **.02**^1^ |  | -.15 | .08^1^ |
| SES | -.02 | .78^1^ |  | -.06 | .46^1^ |  | **-.19** | **.03**^1^ |
|  | **Mean(SD)** | ***p*-value** |  | **Mean(SD)** | ***p*-value** |  | **Mean(SD)** | ***p*-value** |
| Sex |  | .83^2^ |  |  | .79^2^ |  |  | .40^2^ |
| *Male* | 3.9 (.6) |  |  | 27.9 (16.0) |  |  | 35.9 (8.0) |  |
| *Female* | 3.8 (.7) |  |  | 27.1 (16.0) |  |  | 37.0 (9.9) |  |
| Race/ethnicity |  | .65^3^ |  |  | .90^3^ |  |  | **.042^3†^** |
| *Non-Hispanic White* | 3.8 (.6) |  |  | 26.7 (14.7) |  |  | 35.6 (9.9) |  |
| *Black* | 3.9 (.6) |  |  | 28.9 (17.5) |  |  | 38.0 (8.0) |  |
| *Hispanic* | 3.8 (.5) |  |  | 24.6 (14.1) |  |  | 34.0 (8.4) |  |
| *Other Minority* | 3.8 (.7) |  |  | 26.8 (14.5) |  |  | 33.2 (9.8) |  |
| Geographic locale |  | .76^3^ |  |  | .26^3^ |  |  | .83^3^ |
| *Rural* | 3.7 (.9) |  |  | 24.1 (16.4) |  |  | 37.3 (11.6) |  |
| *Suburb* | 3.9 (.5) |  |  | 29.1 (15.6) |  |  | 36.1 (8.6) |  |
| *City* | 3.8 (.6) |  |  | 25.4 (16.5) |  |  | 36.7 (8.3) |  |

Bolded values denote statistical significance at *p* < .05. Superscript letters denote significant statistical groups detected by pairwise Wilcoxon rank sum tests with Benjamini & Hochberg FDR corrections. Abbreviations: SES, socioeconomic status; zBMI, standardized body mass index

^1^Pearson correlation

^2^Wilcoxon rank sum test

^3^Kruskal-Wallis rank sum test

**^†^**No significant pair-wise comparisons

**Supplemental Table 5.** Associations between α-diversity indexes and dietary variables using general linear models adjusting for zBMI, sex, race/ethnicity, geographic locale, and SES (n=136).

|  | **Shannon** | | |  | **Inverse Simpson** | | |  | | **Faith PD** | | |
| --- | --- | --- | --- | --- | --- | --- | --- | --- | --- | --- | --- | --- |
| **Variable (*n* missing)** | **β^1^** | **SE** | ***p*-value** |  | **β^1^** | **SE** | ***p*-value** |  | **β^1^** | | **SE** | ***p*-value** |
| Overall diet quality (2) | .08 | .17 | .35 |  | .14 | 4.47 | .10 |  | .03 | | 2.49 | .70 |
| Skipping breakfast (2) | .06 | .07 | .51 |  | .02 | 1.91 | .81 |  | .14 | | 1.05 | .12 |
| Eating out (3) | -.14 | .08 | .13 |  | -.14 | 2.16 | .12 |  | -.14 | | 1.20 | .12 |
| Whole grains (2) | -.11 | .08 | .24 |  | **-.17** | **2.17** | **.049** |  | -.02 | | 1.22 | .78 |
| Fruits (2) | .07 | .07 | .43 |  | <.01 | 1.90 | .99 |  | **.17** | | **1.03** | **.045** |
| Vegetables (1) | .13 | .08 | .14 |  | .11 | 2.00 | .21 |  | .09 | | 1.10 | .28 |
| Dairy/cheese (4) | .09 | .07 | .32 |  | .04 | 1.88 | .65 |  | .10 | | 1.03 | .24 |
| Meat (2) | -.11 | .08 | .24 |  | -.09 | 2.13 | .32 |  | -.15 | | 1.16 | .08 |
| Processed meats (3) | **-.19** | **.08** | **.03** |  | **-.23** | **2.05** | **.01** |  | -.16 | | 1.15 | .06 |
| Fried foods (2) | -.07 | .07 | .43 |  | -.05 | 1.94 | .60 |  | .01 | | 1.07 | .90 |
| Fatty snacks (3) | -.10 | .08 | .25 |  | -.10 | 2.20 | .27 |  | -.12 | | 1.21 | .17 |
| Fats/oils (4) | .07 | .07 | .46 |  | .02 | 1.82 | .80 |  | .08 | | 1.01 | .38 |
| Fatty sweet products (3) | -.06 | .08 | .50 |  | -.09 | 2.02 | .29 |  | -.09 | | 1.10 | .28 |
| Sodas/sugary drinks (4) | -.05 | .07 | .59 |  | -.02 | 1.93 | .85 |  | -.02 | | 1.06 | .82 |

Bolded values denote statistical significance at *p* < .05.

^1^Standardized regression coefficient

**Supplemental Table 6.** Associations between study variables and abundance of taxa at the phylum, family, and genus levels in multivariate MaAslin2 models adjusting for zBMI, sex, race/ethnicity, geographic locale, and SES.

| **Variable** | **Phylum** | **Family** | **Genus** | **MaAsLin2 coefficient** | **stderr** | ***p*-value** | ***q*-value^1^** |
| --- | --- | --- | --- | --- | --- | --- | --- |
| Diet Quality | Firmicutes | *Lachnospiraceae* | *Anaerostipes* | 0.86 | 0.25 | <0.001 | 0.025 |
| Skipping Breakfast | Actinobacteriota | *—* | *—* | -0.24 | 0.11 | 0.027 | 0.113 |
|  | Actinobacteriota | *Bifidobacteriaceae* | *—* | -0.38 | 0.15 | 0.013 | 0.106 |
|  | Actinobacteriota | *Bifidobacteriaceae* | *Bifidobacterium* | -0.48 | 0.13 | 0.000 | 0.008 |
|  | Firmicutes | *Lachnospiraceae* | *Anaerostipes* | -0.31 | 0.11 | 0.004 | 0.057 |
|  | Firmicutes | *Lachnospiraceae* | *Fusicatenibacter* | -0.34 | 0.11 | 0.001 | 0.040 |
|  | Verrucomicrobiota | *Akkermansiaceae* | *—* | 0.53 | 0.19 | 0.005 | 0.066 |
|  | Verrucomicrobiota | *Akkermansiaceae* | *Akkermansia* | 0.55 | 0.20 | 0.005 | 0.060 |
| Eating Out | Bacteroidota | *Porphyromonadaceae* | *—* | -0.63 | 0.25 | 0.010 | 0.075 |
|  | Bacteroidota | *Porphyromonadaceae* | *Porphyromonas* | -0.75 | 0.26 | 0.004 | 0.060 |
|  | Firmicutes | *Lachnospiraceae* | *Anaerostipes* | -0.32 | 0.12 | 0.008 | 0.091 |
|  | Firmicutes | *Ezakiella* | *—* | -0.85 | 0.27 | 0.002 | 0.032 |
|  | Proteobacteria | *—* | *—* | -0.41 | 0.18 | 0.020 | 0.110 |
| Whole Grains | Firmicutes | *Erysipelotrichaceae* | *—* | -0.44 | 0.18 | 0.016 | 0.106 |
|  | Firmicutes | *Streptococcaceae* | *—* | -0.59 | 0.21 | 0.005 | 0.057 |
|  | Firmicutes | *Streptococcaceae* | *Streptococcus* | -0.63 | 0.20 | 0.002 | 0.043 |
| Fruits | Bacteroidota | *Prevotellaceae* | *—* | 0.62 | 0.19 | 0.002 | 0.030 |
|  | Bacteroidota | *Prevotellaceae* | *Prevotella* | 0.54 | 0.20 | 0.007 | 0.091 |
|  | Firmicutes | *—* | *—* | -0.09 | 0.04 | 0.018 | 0.088 |
|  | Firmicutes | *Lactobacillaceae* | *—* | 0.62 | 0.26 | 0.019 | 0.117 |
|  | Firmicutes | *Veillonellaceae* | *—* | -0.37 | 0.14 | 0.010 | 0.073 |
|  | Verrucomicrobiota | *Akkermansiaceae* | *Akkermansia* | -0.47 | 0.19 | 0.014 | 0.123 |
| Vegetables | Actinobacteriota | *—* | *—* | -0.23 | 0.11 | 0.034 | 0.139 |
|  | Actinobacteriota | *Actinomycetaceae* | *—* | 0.46 | 0.21 | 0.025 | 0.147 |
|  | Firmicutes | *Lachnospiraceae* | *Anaerostipes* | 0.30 | 0.11 | 0.006 | 0.074 |
|  | Firmicutes | *Finegoldia* | *—* | 0.44 | 0.19 | 0.024 | 0.147 |
|  | Proteobacteria | *—* | *—* | -0.68 | 0.16 | <0.001 | <0.001 |
| Dairy | Firmicutes | *Erysipelotrichaceae* | *—* | 0.52 | 0.16 | 0.001 | 0.035 |
|  | Firmicutes | *Lactobacillaceae* | *—* | 1.03 | 0.27 | <0.001 | 0.010 |
|  | Firmicutes | *Lactobacillaceae* | *Lactobacillus* | 0.93 | 0.27 | <0.001 | 0.021 |
| Meat | Firmicutes | *Erysipelotrichaceae* | *—* | -0.65 | 0.18 | <0.001 | 0.016 |
|  | Firmicutes | *Erysipelotrichaceae* | *Turicibacter* | -0.54 | 0.23 | 0.019 | 0.149 |
|  | Firmicutes | *Oscillospiraceae* | *Colidextribacter* | -0.42 | 0.16 | 0.009 | 0.090 |
| Processed Meat | Firmicutes | *Lachnospiraceae* | *Roseburia* | -0.52 | 0.19 | 0.005 | 0.075 |
|  | Proteobacteria | *—* | *—* | 0.44 | 0.17 | 0.010 | 0.055 |
| Fried Foods | Firmicutes | *Lachnospiraceae* | *Anaerostipes* | -0.33 | 0.11 | 0.002 | 0.044 |
| Fatty Snacks | Actinobacteriota | *Bifidobacteriaceae* | — | 0.42 | 0.17 | 0.013 | 0.100 |
|  | Firmicutes | *Peptostreptococcaceae* | *—* | 0.55 | 0.15 | <0.001 | 0.017 |
|  | Firmicutes | *Peptostreptococcaceae* | *Romboutsia* | 0.52 | 0.19 | 0.005 | 0.075 |
| Fats/Oils | Firmicutes | *Ezakiella* | *—* | 0.61 | 0.23 | 0.008 | 0.076 |
| Sweets | Bacteroidota | *Porphyromonadaceae* | *—* | -0.58 | 0.23 | 0.013 | 0.094 |
|  | Bacteroidota | *Porphyromonadaceae* | *Porphyromonas* | -0.67 | 0.24 | 0.006 | 0.076 |
|  | Firmicutes | *Erysipelatoclostridiaceae* | *—* | 0.37 | 0.15 | 0.018 | 0.107 |
|  | Firmicutes | *Anaerococcus* | *—* | -0.48 | 0.21 | 0.023 | 0.118 |
|  | Firmicutes | *Anaerovoracaceae* | *—* | -0.62 | 0.18 | <0.001 | 0.018 |
|  | Firmicutes | *Fenollaria* | *—* | -0.89 | 0.23 | <0.001 | 0.014 |
|  | Firmicutes | *Peptoniphilus* | *—* | -0.50 | 0.22 | 0.023 | 0.118 |
|  | Firmicutes | *Veillonellaceae* | *—* | -0.35 | 0.15 | 0.021 | 0.114 |
| Soda/Sugary Drinks | Firmicutes | *Lachnospiraceae* | *Fusicatenibacter* | -0.28 | 0.10 | 0.007 | 0.075 |
| zBMI | Bacteroidota | *Porphyromonadaceae* | *Porphyromonas* | 0.28 | 0.12 | 0.016 | 0.133 |
|  | Bacteroidota | *Rikenellaceae* | *Alistipes* | -0.15 | 0.06 | 0.012 | 0.115 |
|  | Firmicutes | *Veillonellaceae* | — | 0.17 | 0.07 | 0.015 | 0.102 |
|  | Proteobacteria | *—* | — | -0.28 | 0.08 | <0.001 | 0.006 |
| Black Race | Actinobacteriota | *—* | *—* | 0.43 | 0.20 | 0.032 | 0.130 |
|  | Actinobacteriota | *Actinomycetaceae* | *—* | 1.21 | 0.38 | 0.001 | 0.026 |
|  | Bacteroidota | *Tannerellaceae* | *—* | -0.84 | 0.31 | 0.008 | 0.063 |
|  | Bacteroidota | *Tannerellaceae* | *Parabacteroides* | -0.88 | 0.37 | 0.017 | 0.133 |
|  | Firmicutes | *Lactobacillaceae* | *Lactobacillus* | 1.20 | 0.50 | 0.017 | 0.133 |
|  | Firmicutes | *Christensenellaceae* | *—* | 0.61 | 0.27 | 0.025 | 0.140 |
|  | Firmicutes | *Lachnospiraceae* | *—* | -0.40 | 0.13 | 0.002 | 0.032 |
|  | Firmicutes | *Lachnospiraceae* | *Roseburia* | -0.97 | 0.32 | 0.002 | 0.040 |
|  | Firmicutes | *Ezakiella* | *—* | 1.02 | 0.46 | 0.027 | 0.140 |
|  | Proteobacteria | *—* | *—* | -0.87 | 0.29 | 0.003 | 0.034 |
|  | Proteobacteria | *Sutterellaceae* | *—* | -0.64 | 0.29 | 0.025 | 0.140 |
|  | Verrucomicrobiota | *—* | *—* | 0.98 | 0.34 | 0.004 | 0.035 |
|  | Verrucomicrobiota | *Akkermansiaceae* | *—* | 1.09 | 0.35 | 0.002 | 0.027 |
|  | Verrucomicrobiota | *Akkermansiaceae* | *Akkermansia* | 1.51 | 0.36 | <0.001 | 0.002 |
| Hispanic Ethnicity | Actinobacteriota | *—* | *—* | 0.73 | 0.31 | 0.018 | 0.078 |
|  | Desulfobacterota | — | — | -1.06 | 0.45 | 0.018 | 0.078 |
|  | Desulfobacterota | *Desulfovibrionaceae* | *—* | -1.51 | 0.46 | 0.001 | 0.026 |
|  | Firmicutes | *Erysipelotrichaceae* | *Turicibacter* | -1.77 | 0.58 | 0.002 | 0.040 |
|  | Firmicutes | *Clostridiaceae* | *Clostridium* sensu stricto 1 | -1.35 | 0.51 | 0.008 | 0.088 |
|  | Firmicutes | *Lachnospiraceae* | NK4A136 group | -1.29 | 0.38 | <0.001 | 0.021 |
|  | Firmicutes | *Anaerococcus* | — | -1.81 | 0.59 | 0.002 | 0.030 |
|  | Firmicutes | *Fenollaria* | — | -1.76 | 0.65 | 0.006 | 0.061 |
|  | Firmicutes | *Finegoldia* | — | -1.46 | 0.54 | 0.007 | 0.062 |
| Other Racial or Ethnic Minority | Actinobacteriota | *—* | — | 0.97 | 0.35 | 0.005 | 0.040 |
|  | Actinobacteriota | *Actinomycetaceae* | — | 1.59 | 0.66 | 0.016 | 0.103 |
|  | Bacteroidota | *Tannerellaceae* | *—* | -1.44 | 0.55 | 0.009 | 0.067 |
|  | Firmicutes | *Lachnospiraceae* | NK4A136 group | -1.25 | 0.44 | 0.004 | 0.055 |
|  | Firmicutes | *Oscillospiraceae* | *Colidextribacter* | -1.12 | 0.47 | 0.017 | 0.133 |
|  | Firmicutes | *Ruminococcaceae* | *Faecalibacterium* | -0.65 | 0.28 | 0.020 | 0.149 |
|  | Firmicutes | *Ruminococcaceae* | *Incertae Sedis* | -0.83 | 0.34 | 0.017 | 0.133 |
|  | Proteobacteria | *Sutterellaceae* | *—* | -1.37 | 0.50 | 0.006 | 0.061 |
|  | Verrucomicrobiota | *—* | *—* | 2.09 | 0.59 | <0.001 | 0.006 |
|  | Verrucomicrobiota | *Akkermansiaceae* | *—* | 1.83 | 0.61 | 0.003 | 0.032 |
|  | Verrucomicrobiota | *Akkermansiaceae* | *Akkermansia* | 2.72 | 0.63 | <0.001 | 0.002 |
| Female | Firmicutes | *Erysipelotrichaceae* | *—* | 0.54 | 0.23 | 0.020 | 0.118 |
|  | Firmicutes | *Lactobacillaceae* | *—* | 1.55 | 0.38 | <0.001 | 0.005 |
|  | Firmicutes | *Lactobacillaceae* | *Lactobacillus* | 1.30 | 0.38 | <0.001 | 0.021 |
|  | Firmicutes | *Streptococcaceae* | *—* | 0.95 | 0.27 | <0.001 | 0.019 |
|  | Firmicutes | *Streptococcaceae* | *Streptococcus* | 0.62 | 0.26 | 0.017 | 0.133 |
|  | Firmicutes | *Lachnospiraceae* | *[Ruminococcus] gauvreauii* | -0.66 | 0.24 | 0.007 | 0.076 |
|  | Firmicutes | *Lachnospiraceae* | *Lachnoclostridium* | 0.38 | 0.15 | 0.011 | 0.108 |
|  | Verrucomicrobiota | *Akkermansiaceae* | *—* | 0.80 | 0.27 | 0.003 | 0.033 |
| Suburb | Bacteroidota | *—* | *—* | -0.50 | 0.18 | 0.006 | 0.040 |
|  | Bacteroidota | *Bacteroidaceae* | *Bacteroides* | -1.02 | 0.27 | <0.001 | 0.007 |
|  | Bacteroidota | *Marinifilaceae* | *—* | -0.95 | 0.41 | 0.021 | 0.122 |
|  | Firmicutes | *Erysipelatoclostridiaceae* | *—* | 1.08 | 0.34 | 0.002 | 0.026 |
|  | Firmicutes | *Erysipelotrichaceae* | *—* | -0.80 | 0.36 | 0.027 | 0.140 |
|  | Firmicutes | *Lactobacillaceae* | *—* | -1.54 | 0.60 | 0.010 | 0.074 |
|  | Firmicutes | *Lactobacillaceae* | *Lactobacillus* | -1.51 | 0.61 | 0.013 | 0.119 |
|  | Firmicutes | *Clostridiaceae* | *Clostridium* sensu stricto 1 | -1.14 | 0.40 | 0.004 | 0.055 |
|  | Firmicutes | *Lachnospiraceae* | *—* | 0.57 | 0.16 | <0.001 | 0.019 |
|  | Firmicutes | *Lachnospiraceae* | *Agathobacter* | 0.93 | 0.28 | <0.001 | 0.021 |
|  | Firmicutes | *Lachnospiraceae* | *Anaerostipes* | 0.95 | 0.25 | <0.001 | 0.005 |
|  | Firmicutes | *Lachnospiraceae* | *Fusicatenibacter* | 0.63 | 0.24 | 0.008 | 0.088 |
|  | Firmicutes | *Butyricicoccaceae* | *—* | 1.15 | 0.35 | 0.001 | 0.026 |
|  | Firmicutes | *Butyricicoccaceae* | *Butyricicoccus* | 1.07 | 0.35 | 0.002 | 0.040 |
|  | Firmicutes | *Oscillospiraceae* | *—* | -0.61 | 0.23 | 0.008 | 0.065 |
|  | Firmicutes | *Oscillospiraceae* | UCG-005 | -1.16 | 0.40 | 0.003 | 0.049 |
|  | Firmicutes | *Ruminococcaceae* | *—* | 0.65 | 0.18 | <0.001 | 0.019 |
|  | Firmicutes | *Ruminococcaceae* | *Faecalibacterium* | 0.80 | 0.19 | <0.001 | 0.002 |
|  | Firmicutes | *Anaerococcus* | *—* | 1.25 | 0.46 | 0.007 | 0.062 |
|  | Firmicutes | *Finegoldia* | *—* | 1.38 | 0.43 | 0.001 | 0.026 |
|  | Firmicutes | *Peptostreptococcaceae* | *Romboutsia* | -0.96 | 0.38 | 0.011 | 0.108 |
|  | Proteobacteria | *Sutterellaceae* | *—* | 0.77 | 0.35 | 0.026 | 0.140 |
|  | Verrucomicrobiota | *—* | *—* | -1.11 | 0.41 | 0.007 | 0.040 |
|  | Verrucomicrobiota | *Akkermansiaceae* | *—* | -1.11 | 0.42 | 0.008 | 0.065 |
|  | Verrucomicrobiota | *Akkermansiaceae* | *Akkermansia* | -1.34 | 0.44 | 0.002 | 0.040 |
| City | Actinobacteriota | *Bifidobacteriaceae* | *—* | 1.06 | 0.42 | 0.011 | 0.078 |
|  | Bacteroidota | *Bacteroidaceae* | *Bacteroides* | -1.43 | 0.34 | 0.000 | 0.002 |
|  | Bacteroidota | *Marinifilaceae* | *—* | -1.17 | 0.51 | 0.021 | 0.122 |
|  | Firmicutes | *Erysipelatoclostridiaceae* | *—* | 1.24 | 0.42 | 0.003 | 0.036 |
|  | Firmicutes | *Lactobacillaceae* | *—* | -1.74 | 0.74 | 0.018 | 0.113 |
|  | Firmicutes | *Lactobacillaceae* | *Lactobacillus* | -2.25 | 0.75 | 0.003 | 0.042 |
|  | Firmicutes | *Streptococcaceae* | *—* | 1.45 | 0.54 | 0.007 | 0.062 |
|  | Firmicutes | *Christensenellaceae* | *—* | -1.29 | 0.41 | 0.001 | 0.026 |
|  | Firmicutes | *Christensenellaceae* | CR-7 group | -1.19 | 0.43 | 0.005 | 0.062 |
|  | Firmicutes | *Lachnospiraceae* | *—* | 0.72 | 0.20 | <0.001 | 0.019 |
|  | Firmicutes | *Lachnospiraceae* | *Agathobacter* | 1.09 | 0.34 | 0.001 | 0.033 |
|  | Firmicutes | *Lachnospiraceae* | *Anaerostipes* | 0.95 | 0.30 | 0.002 | 0.038 |
|  | Firmicutes | *Lachnospiraceae* | *Blautia* | 0.54 | 0.17 | 0.002 | 0.038 |
|  | Firmicutes | *Butyricicoccaceae* | *—* | 1.32 | 0.43 | 0.002 | 0.032 |
|  | Firmicutes | *Butyricicoccaceae* | *Butyricicoccus* | 1.28 | 0.43 | 0.003 | 0.046 |
|  | Firmicutes | *Oscillospiraceae* | *—* | -0.92 | 0.29 | 0.001 | 0.026 |
|  | Firmicutes | *Oscillospiraceae* | UCG-002 | -0.88 | 0.37 | 0.016 | 0.133 |
|  | Firmicutes | *Oscillospiraceae* | UCG-005 | -1.70 | 0.49 | <0.001 | 0.019 |
|  | Firmicutes | *Ruminococcaceae* | *—* | 0.72 | 0.23 | 0.002 | 0.026 |
|  | Firmicutes | *Ruminococcaceae* | *Faecalibacterium* | 0.96 | 0.24 | <0.001 | 0.003 |
|  | Firmicutes | *Veillonellaceae* | *Dialister* | -1.42 | 0.49 | 0.004 | 0.055 |
|  | Proteobacteria | *Sutterellaceae* | *—* | 1.18 | 0.43 | 0.006 | 0.059 |
|  | Verrucomicrobiota | *—* | *—* | -2.43 | 0.50 | <0.001 | <0.001 |
|  | Verrucomicrobiota | *Akkermansiaceae* | *—* | -2.36 | 0.52 | <0.001 | 0.001 |
|  | Verrucomicrobiota | *Akkermansiaceae* | *Akkermansia* | -3.27 | 0.54 | <0.001 | <0.001 |
| Socioeconomic Status | Actinobacteriota | *Bifidobacteriaceae* | *—* | -0.34 | 0.14 | 0.016 | 0.103 |
|  | Actinobacteriota | *Eggerthellaceae* | *—* | -0.39 | 0.15 | 0.010 | 0.074 |
|  | Desulfobacterota | *—* | *—* | -0.40 | 0.15 | 0.008 | 0.040 |
|  | Desulfobacterota | *Desulfovibrionaceae* | *—* | -0.44 | 0.15 | 0.004 | 0.039 |
|  | Firmicutes | *[Eubacterium] coprostanoligenes* group | *—* | -0.44 | 0.13 | <0.001 | 0.019 |
|  | Firmicutes | *Oscillospiraceae* | UCG-002 | -0.35 | 0.12 | 0.005 | 0.062 |

**^1^** Benjamini-Hochberg corrected p-value with a 15% false discover rate.

**Supplemental Figure 1.** Study flow chart of the participants that were included in the analysis. Of the 288 adolescents enrolled into Wave 1 of the Adolescent Diet Study, 3 participants were excluded for not submitting a REAP-S, 148 participants were excluded for not submitting a fecal sample, and 1 participant was excluded due to insufficient sequencing depth resulting in a final sample size of n = 136 adolescents included in the analyses.

**Supplemental Figure 2.** Relative abundance (RA) of the five most abundant bacterial phyla. Each vertical bar represents the relative abundance determined in a stool sample from one study participant (n = 136).


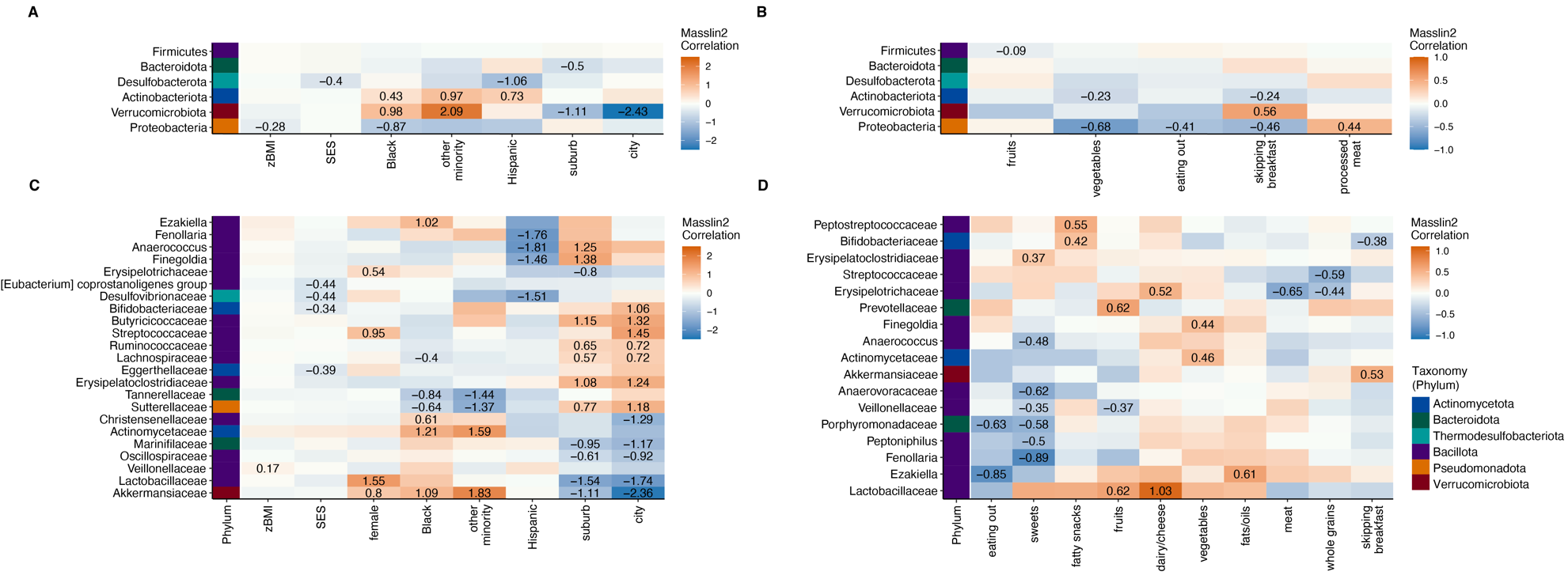


**Supplemental Figure 3.** Associations between study variables and taxa abundance using Microbiome Multivariable Associations with Linear Models (MaAsLin2, package on R). Only significant associations are included in the heatmaps for (A) phyla abundance and sociodemographic variables, (B) phyla abundance and dietary variables, (C) family abundance and sociodemographic variables, and (D) family abundance and dietary variables. All MaAsLin2 models multi-adjusted for *sex* (female/male), *race/ethnicity* (Non-Hispanic White[*reference*], Black, Hispanic, other minority), *standardized body mass index* (zBMI), *socioeconomic status* (SES), and *locale* (rural[*reference*], suburb, city). The MaAsLin2 correlation coefficient (effect size) is shown only for significant associations after Benjamini–Hochberg correction for multiple comparisons with a 15% false discovery rate threshold. Taxa are displayed on the y-axis and are color-coded by phyla.
